# Primary Epstein-Barr virus infection with polyradiculitis: a case report

**DOI:** 10.1186/1471-2377-13-96

**Published:** 2013-07-23

**Authors:** Tilmann Hottenrott, Sebastian Rauer, Jochen Bäuerle

**Affiliations:** 1 Department of Neurology, University Hospital Freiburg, Breisacher Str. 64, 79106, Freiburg, Germany

**Keywords:** Primary Epstein-Barr virus (EBV) infection, Infectious mononucleosis, Polyradiculitis

## Abstract

**Background:**

Nervous system complications of primary Epstein-Barr virus (EBV) infection in adults are rare, but may occur with encephalitis, meningitis, myelitis, cranial and peripheral neuropathies, or radiculitis.

**Case presentation:**

We describe an immune competent adult with a primary EBV infection complicated by lumbosacral polyradiculitis with pure radicular pain. Prior to the onset of radicular pain the 35-year-old woman had been suffering from infectious mononucleosis misdiagnosed for streptococcal tonsillitis. The diagnosis of primary EBV infection associated polyradiculitis was proven by serology and PCR in serum and CSF. Under initially started empiric therapy with intravenous acyclovir and analgesics the patient completely recovered within a few days.

**Conclusion:**

This case report highlights that EBV should be taken into consideration in the diagnostic work up of radicular pain syndromes, even in immune competent adults. There is no approved causal therapy for EBV infections. In accordance with our case, observations based on a few patients with EBV and nervous system involvement suggest, that acyclovir treatment might be associated a with better course. However, prospective randomized controlled trials addressing the question of the effectiveness of acyclovir in patients with primary EBV infection and neurological complications are lacking.

## Background

Nervous system complications of primary Epstein-Barr virus (EBV) infection in adults are rare (< 8%) [1,2], but may occur with encephalitis, meningitis, myelitis, cranial and peripheral neuropathies, or radiculitis [3,4]. Here we describe an immune competent adult with a primary EBV infection complicated by lumbosacral polyradiculitis with pure radicular pain.

## Case presentation

A 35-year-old woman presented with five days of nightly accentuated burning pain in both legs leading to massive sleep disturbance. The pain was predominantly located in the dorsal thighs and radiated to the calves. She reported some relief through walking around at night and showering her legs with hot water. Back pain, gait or sensory disturbances and bladder dysfunction were denied. Nonsteroidal anti-inflammatory drugs did not alleviate pain, neither did pramipexole, which had been prescribed for suspected restless leg syndrome. One week before she had had a sore throat and had been clinically diagnosed for streptococcal tonsillitis and treated empirically with penicillin. Her past medical and family history was unremarkable. Clinical examination revealed no focal-neurologic signs. There were no sensory deficits and deep tendon reflexes were symmetrically normoactive. Furthermore, we found no palpable lymph node swelling and no signs of pharyngitis. Motor and sensory nerve conduction studies were normal, as were F-responses of the tibial nerve. Abdominal Ultrasound demonstrated a slightly enlarged spleen. Blood sampling showed relative lymphocytosis, and elevated liver enzymes. Cerebrospinal fluid analysis revealed 18 white blood cells/μl (90% lymphocytes, 10% monocytes), 83mg/dl protein, and blood–brain-barrier dysfunction (albumin CSF/serum quotient 12.5 × 10^-3^) without intrathecal immunoglobulin synthesis. Virological studies detected EBV VCA IgG and IgM in serum, whereas EBV EBNA-1 IgG was negative. Moreover, EBV-PCR (RealStar® EBV PCR Kit 1.0, altona Diagnostics GmbH, Germany) was positive in blood and CSF (1000 copies/ml each) suggesting primary EBV infection involving the nervous system. Further laboratory investigations were not indicative for infections with streptococcus, borrelia, syphilis, HIV, hepatitis virus A, B or C (serological tests in blood, borrelia and syphilis additionally in CSF), VZV, HSV or enteroviruses (VZV- and HSV-IgG-specificity-indices and PCR in CSF). Under initially started empiric therapy with intravenous acyclovir for possible VZV or HSV radiculitis and analgesic treatment with oral tramadol the patient completely recovered within a few days. After receiving negative results for VZV and HSV, acyclovir was ceased on day six.

## Conclusion

To our knowledge, we report the first case of primary Epstein-Barr virus infection complicated by polyradiculitis with pure radicular pain. Several studies described nervous system manifestations of infectious mononucleosis with a broad spectrum of neurologic deficits [3-5]. This case may represent the benign variant of EBV associated polyradiculitis. Therefore EBV should be taken into consideration in the diagnostic work up of radicular pain syndromes, even in immune competent individuals.

In order to establish definite diagnosis serological testing is based on measuring antibodies directed against the EBV nuclear antigen (EBNA) and the Epstein-Barr viral capsid antigen (VCA). Polymerase chain reaction may be a useful tool for the early diagnosis of infectious mononucleosis [5]. Otherwise, especially in profoundly immune compromised subjects interpretation of EBV PCR can be challenging, since co-infections with other pathogens are common in these patients [6].

The pathogenesis of EBV nervous system infection is not fully understood, besides direct viral invasion indirect immune effects are discussed as potential mechanisms [7]. A better pathophysiological understanding might have therapeutical implications, as there is no available evidence-based therapy for EBV infection. Interestingly, Bathoorn et al. observed in a small retrospective case series, that, in accordance with our case, all patients (n = 5) with primary EBV infection and neurological complications treated with acyclovir recovered fully, whereas 3 of 4 without antiviral treatment developed a complicated course [4]. We are aware of the limited significance of this case report since we are not able to estimate the effect of acyclovir on our patient. With regard to the mild form of EBV associated polyradiculitis, the clinical course might have been uncomplicated even if acyclovir had not been given. However, a prospective randomized controlled trial addressing the question of the effectiveness of intravenous acyclovir in patients with primary EBV infection and neurological complications is lacking.

## Consent

Written informed consent was obtained from the patient for publication of this case report.

## Competing interests

The authors have no competing interests to declare.

## Authors’ contributions

TH has analyzed the case and drafted the manuscript. All authors have made substantial contributions to interpretation of the case; they have revised the manuscript critically for important intellectual content and have given their final approval of the version to be published.

## Pre-publication history

The pre-publication history for this paper can be accessed here:

http://www.biomedcentral.com/1471-2377/13/96/prepub
